# Ovine Progressive Pneumonia: Diagnosis and Seroprevalence in the South of Sonora, Mexico

**DOI:** 10.1155/2021/6623888

**Published:** 2021-02-04

**Authors:** Mercedes Yannin Borquez Cuevas, Juan Francisco Hernández Chávez, Betsy Armenta Leyva, Jesús Raymundo Cedillo Cobián, Ramón Miguel Molina Barrios

**Affiliations:** Departamento de Ciencias Agronómicas y Veterinarias, Instituto Tecnológico de Sonora, Antonio Caso S/N C.P. 85093 Cd. Obregón, Sonora, Mexico

## Abstract

Ovine progressive pneumonia (OPP) is the most severe presentation of small ruminant lentivirus (SRLV) infection known as Maedi-Visna. Serological evidence in Mexico of the presence of this lentivirus was published in 1986. After that, studies revealed that SRLVs have a broad distribution in Mexico by detecting antibodies or/and molecular tests; however, a descriptive case of the disease has not been published. This work's objective was to describe the diagnosis of a case of OPP through lesion description, serology, and molecular test. The histopathological study showed that lymph follicular hyperplasia, interstitial pneumonia, and smooth muscle hyperplasia were presented. The serological test demonstrated specific antibodies against the Maedi-Visna virus, and PCR analysis demonstrated a positive outcome. These results include the criteria for the diagnosis of OPP. The serological prevalence of this disease is presented, contributing to the knowledge of the ecology of this disease in the world. This work is the first case report of ovine progressive pneumonia in Mexico and evidence of seroprevalence in sheep herds from Sonora, Mexico.

## 1. Introduction

Maedi-Visna virus (MVV) and caprine arthritis encephalitis virus (CAEV) are members of the group of small ruminant lentiviruses (SRLVs) classified in the OIE-World Organization for Animal Health list of notifiable animal diseases. These diseases result in progressive and persistent infections that affect animal health and international trade. Clinical signs may include progressive weight loss, chronic respiratory disease in sheep, or hard udders with reduced milk production [1–3]. Under field conditions, diagnosis of ovine progressive pneumonia includes clinical signs, macroscopic and microscopic lesions, and the detection of specific antibodies against the virus using the AGID test or the ELISA technique; however, etiological detection is best performed by the classical or the real-time version of PCR [4–7]. As a consequence that OPP is a slowly progressive disease, only a small proportion of infected sheep develop pathognomonic lesions; the detection of specific antibodies against the MVV in the serum samples and conclusive diagnosis using molecular techniques are the most valuable methods for the detection of OPP in the herds [6, 8].

In México, serological evidence of the presence of Maedi-Visna in native sheep was previously published [9]. After that, some studies through the detection of antibodies or molecular tests revealed that SRLVs have a wide distribution in small ruminant herds in Mexico [10, 11]; however, a descriptive case of this disease has not been published in Mexico. This work is aimed at describing OPP's diagnosis through macroscopic and microscopic lesion description, serology, and molecular tests. Furthermore, evidence of the prevalence of this disease in this geographic region is presented.

## 2. Case Presentation

**Figure 1:**
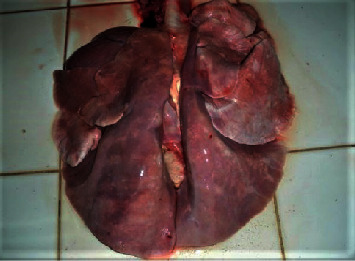
Gross appearance of ovine progressive pneumonia in the lung of a sheep. The affected lung is enlarged and heavy and has no collapse, with rib impressions on the costal surfaces of the diaphragmatic lobes.

A Pelibuey breed sheep, female, two years of age with a history of clinical respiratory problems and chronic wasting, was referred to the Instituto Tecnológico de Sonora's Pathology Laboratory. Progressive dyspnea, a markedly increased respiratory rate with panting after gathering, frothy nasal discharge from both nostrils for some weeks, and chronic wasting were characteristics in this animal. The physical examination showed a body condition score of 2/5, with alopecic areas on the back, extreme foot growth, and moderate dehydration. Enrofloxacin with bromhexine treatment was parenterally administered a dose of 5.0 mg/kg per day for five days, but no signs of improvement were obtained. The sheep were euthanized by an overdose of barbiturate followed by bleed-out. At necropsy, the lung lobes were not collapsed and enlarged, with rib imprints, rubbery consistency, and meaty appearance primarily on diaphragmatic lobes (Figure 1); mediastinal lymph nodes were enlargedly edematous, and a dilated right ventricle and serous atrophy of fat were apparent in the heart. During the necropsy procedure, tissue samples were collected and fixed in 10% neutral-buffered formalin for 48 h at room temperature; after that, tissues were run by routine histology processing with inclusion in paraffin wax and stained with hematoxylin and eosin (HE); after that, they were examined using light microscopy (Olympus Cx-41).

**Figure 2:**
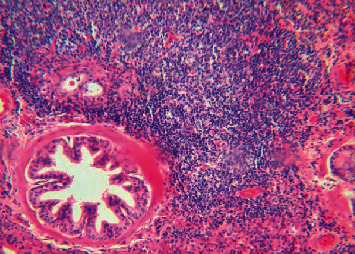
Ovine progressive pneumonia: peribronchiolar lymphoid hyperplasia with formation of prominent well-defined lymphoid nodules and smooth muscle hyperplasia of the bronchiolar wall (H&E, 100x).

**Figure 3:**
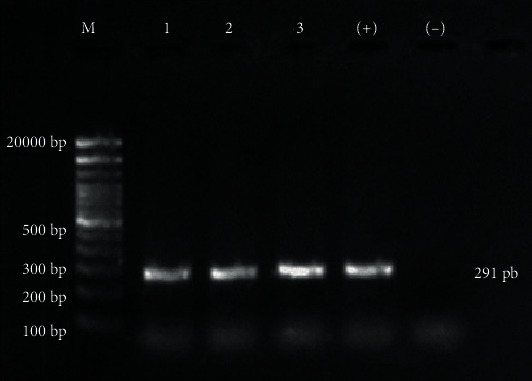
Detection of Maedi-Visna DNA from lung tissue of a sheep by PCR to determine the region of DNA repetition: lane M: 100 pb DNA ladder; lanes 1-3: positive magnification (291 pb); lane (+): positive control; and lane (-): negative control.

Histopathological examination revealed severe lymphoproliferative pneumonia with peribronchiolar and perivascular lymphoid follicles, hyperplasia of bronchioles' smooth muscle, and suppurative bronchitis in the lung sections (Figure 2); apparent on-demand moderate lymphoid hypertrophy in lymph node sections; and mild-moderate congestion with moderate perivascular lymphoid infiltrates in the liver section. Besides, the positive detection of specific antibodies against the virus using the AGID test in serum was detected. Nucleic acid extraction from the lung section was done on a Taco system (taco™, GeneReach Biotech, USA) using a Taco preloaded DNA/RNA extraction kit (GeneReach Biotech, USA) following the manufacturer's instructions. After that, the detection of MV provirus in lung sections by final-point PCR was performed to amplify the MV provirus's LTR sequence, as previously described [12]. In brief, forward (5-TGACACAGCAAAT GTAACCGCAAG-3) and reverse (5-CCA CGTTGGGCGCCAGCTGCGAGA-3) primers were used to amplify a 291 bp fragment of the LTR region. A temperature cycling procedure consisted of denaturation at 94°C for 30 s, annealing at 58°C for 30 s, and extension at 72°C for 40 s. The cycling was repeated 35 times. The PCR products were analyzed on a 1.6% agarose gel electrophoresis (AGR-LE-100, Axygen) and stained with ethidium bromide (46067 Fluka). Amplification of 291 bp fragment is displayed (Figure 3).

Since no previous reports of the prevalence of OPP in the state of Sonora, Mexico, have been published, a cross-sectional study was conducted to determine the prevalence of this disease in the northwest of Mexico. From 30 herds situated at southern Sonora State, Mexico, 450 serum samples from individual sheep were collected and tested through the agar gel immunodiffusion (AGID) test. The results showed that a true prevalence of OPP was 3.7%, and 26.6% of flocks had at least one seropositive animal. In addition, 47% (8/17) of positive animals were recently introduced to local herds, and 76% (13/17) of positive animals were older than 2.5 years.

## 3. Discussion

In Mexico, very scarce data is available regarding SRLV infection in the small ruminant. The absence of a national control program and not active surveillance programs are the main risk to the widespread disease. Moreover, free trade of live small ruminants from different regions and countries where the disease has been reported could be the leading cause of dissemination [1].

Gross findings in pulmonary lobes were similar to lesions described in previous reports [5, 8, 12]. Likewise, histopathological findings showed that lymph follicular hyperplasia, interstitial pneumonia, and smooth muscle hyperplasia characteristic of the OPP were presented in different lung sections, and they are comparable to previous reports [4, 5, 12].

In accord with the statement, the final diagnosis of OPP is to be made based on a supportive clinical history of the disease, the characteristic lesions at necropsy and histopathological findings; confirmed by serological examinations and molecular techniques [5, 13], results obtained in this study confirmed the presence of a clinical case of OPP in Mexico.

In this serological study, almost half of the positive animals have introduced animals with any epidemiological control. Serological results indicate that Maedi-Visna virus (MVV) is circulating among herds, but at low prevalence compared with reports from Canada (13%) as reported by Fournier et al. [14] or Spain (77%) [15].

## 4. Conclusions

Based on pathological findings, serological results, and molecular detection of MVV, we established that this work is the first report of a clinical presentation of OPP in Mexico.

Based on the serological study, the MVV is flowing among the herds in the south of Sonora State, Mexico. A national or regional program of surveillance is necessary to control this disease.

## Data Availability

The data used to support the findings of this study are included within the article.
